# Association between work-related stress and lifestyle behaviors

**DOI:** 10.1192/j.eurpsy.2025.1981

**Published:** 2025-08-26

**Authors:** M. Bouhoula, S. Ben Fredj, N. Gannoun, I. Kacem, A. Aloui, M. Makhloufi, A. Chouchene, N. Belhaj, Y. Haddad, R. Ghammem, K. Haj Mabrouk, M. Maoua, H. Kalboussi, A. Brahem, S. Mhamdi, L. Bouzgarrou, I. Harrabi, S. Chatti, O. El Maalel

**Affiliations:** 1 occupational medecine, Farhat Hached University Hospital; 2 Faculty of Medicine of Sousse, University of Sousse; 3 Epidemiology Department, Farhat Hached University Hospital; 4 occupational medecine, Sahloul University Hospital; 5 Occupational Medicine Group, Sousse; 6 Epidemiology Department, Fattouma Bourguiba University Hospital, Monastir; 7 occupational medecine, Haj Ali Soua Hospital, ksar hellal, Tunisia

## Abstract

**Introduction:**

Work-related stress significantly impacts employees’ overall health and can lead to unhealthy lifestyle behaviors, such as poor diet, lack of physical activity, and inadequate sleep.

**Objectives:**

This study aimed to explore the potential link between occupational stress and different lifestyle behaviors among workers in different companies in Sousse, Tunisia.

**Methods:**

A cross-sectional study was conducted over a three-year period in Sousse, Tunisia, involving employees from various workplace settings. Data were collected using a pre-established questionnaire that assessed sociodemographic characteristics, health-related behaviors—including sleep quantity and quality, cigarette smoking, alcohol consumption, physical activity (measured with the Global Physical Activity Questionnaire), and eating habits—as well as occupational characteristics. Occupational stress was evaluated using a validated Arabic version of the Karasek scale. Binary logistic regression was employed to calculate adjusted odds ratios with 95% confidence intervals.

**Results:**

The study included 154 participants, predominantly female (56.5%), with a mean age of 39.99 ± 9.91 years. Approximately 34.6% had 11 to 20 years of seniority. Job strain and iso-strain were reported by 31.8% and 25.5% of workers, respectively. Job strain prevalence was observed in 32% of smokers, 41% of alcohol users, 40 % among those who do not meet the recommended levels of physical activity. In terms of sleep quality, 29.9% indicated poor sleep. Notably, our study revealed a significant association between job strain and good sleep quality (aOR=6.14; CI95%:1.72-21.95, p=0.005).

**Conclusions:**

These findings highlight a concerning prevalence of unhealthy lifestyle behaviors among workers in Sousse, Tunisia, with significant associations between occupational stress and sleep quality. Addressing these issues through workplace wellness programs may enhance employee health and overall job satisfaction.

**Disclosure of Interest:**

None Declared

